# Hyaluronic Acid and Large Extracellular Vesicles (EVs) in Synovial Fluid and Plasma of Patients With End-Stage Arthritis: Positive Association of EVs to Joint Pain

**DOI:** 10.1177/19476035241247659

**Published:** 2024-05-10

**Authors:** Anne-Mari Mustonen, Janne Capra, Sanna Oikari, Laura Säisänen, Lauri Karttunen, Petro Julkunen, Petri Lehenkari, Antti Joukainen, Antti Jaroma, Tommi Paakkonen, Tommi Kääriäinen, Heikki Kröger, Petteri Nieminen

**Affiliations:** 1 Institute of Biomedicine, School of Medicine, Faculty of Health Sciences, University of Eastern Finland, Kuopio, Finland; 2 Department of Environmental and Biological Sciences, Faculty of Science, Forestry and Technology, University of Eastern Finland, Joensuu, Finland; 3 Cell and Tissue Imaging Unit, Institute of Biomedicine, School of Medicine, Faculty of Health Sciences, University of Eastern Finland, Kuopio, Finland; 4 Department of Clinical Neurophysiology, Kuopio University Hospital, Kuopio, Finland; 5 Department of Technical Physics, Faculty of Science, Forestry and Technology, University of Eastern Finland, Kuopio, Finland; 6 Department of Rehabilitation, Kuopio University Hospital, Kuopio, Finland; 7 Cancer and Translational Medicine Research Unit, Faculty of Medicine, University of Oulu, Oulu, Finland; 8 Department of Surgery and Medical Research Center, Oulu University Hospital, Oulu, Finland; 9 Pihlajalinna, Kuopio, Finland; 10 Department of Orthopaedics, Traumatology and Hand Surgery, Kuopio University Hospital, Kuopio, Finland; 11 Kuopio Musculoskeletal Research Unit, University of Eastern Finland, Kuopio, Finland

**Keywords:** extracellular vesicles, hyaluronan, osteoarthritis, rheumatoid arthritis, synovial fluid

## Abstract

**Objective:**

Hyaluronic acid (HA) in synovial fluid (SF) contributes to boundary lubrication with altered levels in osteoarthritis (OA) and rheumatoid arthritis (RA). SF extracellular vesicles (EVs) may participate in arthritis by affecting inflammation and cartilage degradation. It remains unknown whether HA and EVs display joint-specific alterations in arthritic SFs.

**Design:**

We investigated the numbers and characteristics of HA-particles and large EVs in SF from knees and shoulders of 8 OA and 8 RA patients and 8 trauma controls, and in plasma from 10 healthy controls and 11 knee OA patients. The plasma and SF HA concentrations were determined with a sandwich-type enzyme-linked sorbent assay, and EVs and HA-particles were characterized from plasma and unprocessed and centrifuged SFs with confocal microscopy. The data were compared according to diagnosis, location, and preanalytical processing.

**Results:**

The main findings were: (1) OA and RA SFs can be distinguished from trauma joints based on the distinctive profiles of HA-particles and large EVs, (2) there are differences in the SF HA and EV characteristics between shoulder and knee joints that could reflect their dissimilar mobility, weight-bearing, and shock absorption properties, (3) EV counts in SF and plasma can positively associate with pain parameters independent of age and body adiposity, and (4) low-speed centrifugation causes alterations in the features of HA-particles and EVs, complicating their examination in the original state.

**Conclusions:**

Arthritis and anatomical location can affect the characteristics of HA-particles and large EVs that may have potential as biomarkers and effectors in joint degradation and pain.

## Introduction

Cell-derived extracellular vesicles (EVs) have recently emerged as potential contributors to inflammation and cartilage degradation in age-related degenerative joint diseases,^1,2^ while their exact roles remain inconclusive. Still, the autoimmune-driven rheumatoid arthritis (RA) displays elevated EV counts in synovial fluid (SF) compared to the less inflammatory osteoarthritis (OA). EVs have been proposed to stimulate pro-inflammatory pathways in synovial joints by transporting inflammatory factors and cartilage-degrading proteinases, and by inducing their production by joint cells. They are also involved in pain processes, have the ability to relieve pain, and they transport bioactive molecules with potential as pain biomarkers.^3^ SF EVs from OA and RA patients are heterogenous in size, and they include larger membrane-bound particles >1000 nm in diameter.^4,5^ The data on the cargo and function of these large EVs are scarce, as they are often routinely discarded by EV isolation techniques focusing on significantly smaller exosomes.^6^ However, large EVs detected in RA SF can associate with autoantigens, be citrullinated, and form bioactive inflammatory immune complexes,^7^ indicating that their potential effects would be significant enough to be included in studies of EVs. All membrane-bound particles irrespective of their size are referred to as EVs in this study.

Among other bioactive molecules, SF EVs can transport hyaluronic acid (HA)—a high-molecular-weight glycosaminoglycan secreted by fibroblast-like synoviocytes.^8^ Total HA concentrations and molecular weight of HA-particles can decrease in the SF of arthritic joints with potential effects on articular cartilage lubrication.^9^ HA also provides immunomodulatory and anti-inflammatory effects, protection of extracellular matrix, and pain relief, and can be administered as intra-articular treatments for arthritis patients,^10^ although its clinical effectiveness and adverse effects remain controversial.^11^ Large HA-containing EVs (HA–EVs) have been reported to be released by several cell types,^8,12^ but their functions in synovial joints remain obscure. Knowledge of the biology of these EVs could contribute to a better understanding of pathogeneses of joint diseases, and they could represent a plausible biomarker candidate or therapeutic target.^2^ Finally, both EVs and HA have great potential in applications to improve cartilage repair, especially when administered in combination.^13^

We have previously characterized HA–EVs in the knee SF of OA, RA, and trauma patients.^5^ This aspect was expanded to study if large EVs and HA-particles would be affected by arthropathies in SF and plasma and if they would associate with physiatric data including functionality and pain. In addition to potentially altered SF EV counts and cargo in joint diseases with low- or high-grade inflammation,^1,2,14^ there are indications that the SF composition of adipokines and fatty acids varies between joints of different anatomical location.^15,16^ The fact that these molecules can be transported in EVs^8,17^ prompted us to investigate, whether there would also be differences in EVs or HA-particles between shoulder and knee joints of orthopedic patients. Moreover, SF is often centrifuged after sampling to remove cells and debris.^7^ However, low-speed centrifugation could also affect the characteristics of the desired research objects, causing a potential confounding factor in the analyses and interpretation of results^18^ and, thus, our secondary aim was to investigate this potential source of error with human SF. The hypotheses of this study were as follows: (1) characteristics of large EVs with HA cargo would differ between OA, RA, and trauma controls, possibly due to different levels of inflammation, (2) there would be higher HA concentrations in knee versus shoulder SF, possibly due to different demands for weight-bearing and shock absorption, (3) the numbers of SF and plasma EVs and HA-particles would associate with joint pain and physical disability, and (4) preanalytical processing of SF would change the characteristics of HA-particles and large EVs.

## Methods

### Ethics, Subjects, and Sampling

The study was approved by the Ethical Committees of the Oulu (decision #29/2011, amendment 2/24/2014) and Kuopio University Hospitals (#79//2013, #73/2016, #140/2017, amendment 8/2020) in compliance with the Helsinki Declaration. The general characteristics of the recruited patients (sex, age, body mass index [BMI]) are represented in **Table 1**. All participants were >18 years of age and provided written informed consent to donate their samples. In substudy I, SF was collected from glenohumeral (*n* = 11) and knee joints (*n* = 13) with sterile needles and syringes during arthroscopic or joint replacement surgery and stored at −70°C. The joints were categorized as trauma controls without history of arthritis (4 shoulders, 4 knees), end-stage OA (4 shoulders, 4 knees), and end-stage seropositive RA (3 shoulders, 5 knees). In substudy II, 10 healthy controls and 11 end-stage knee OA patients who donated SF (OA patients only) and fasting blood samples were recruited. Venous blood was collected using BD Vacutainer K2 EDTA tubes (Becton, Dickinson and Company, Franklin Lakes, NJ, USA) and centrifuged at 2500*g* for 15 min at room temperature (RT) followed by a similar second centrifugation of the upper layers of the supernatant to remove any remaining platelets, and storage at −70°C. In addition to these samples, the total HA concentration was determined from another set of control (*n* = 6), OA (*n* = 18), and RA (*n* = 4) shoulder and knee SF samples collected, stored, and analyzed similarly to the original patients. A part of the knee HA data was previously published.^5^

**Table 1.** General Characteristics of the Subjects (Mean ± SE).

	Control	OA	RA	P Diagnosis
*Substudy I*				
Sex (M/F)	4/4	2/6	1/7	0.402
Age	47 ± 4^a^	61 ± 4^ab^	72 ± 3^b^	0.001
BMI	27.2 ± 1.70^a^	34.9 ± 2.17^b^	24.1 ± 1.29^a^	0.002
*Substudy II*				
Sex (M/F)	5/5	4/7		0.670
Age	32 ± 3^a^	62 ± 2^b^		<0.001
BMI	26.1 ± 0.87^a^	31.7 ± 0.62^b^		0.001

OA = osteoarthritis, RA = rheumatoid arthritis, M = male, F = female, BMI = body mass index; sex ratios were tested with the Fisher’s exact test; means with different superscript letters are significantly different from each other within a row (Kruskal–Wallis ANOVA, Mann–Whitney *U* test).

### Confocal Laser Scanning Microscopy

**Figure 1.**
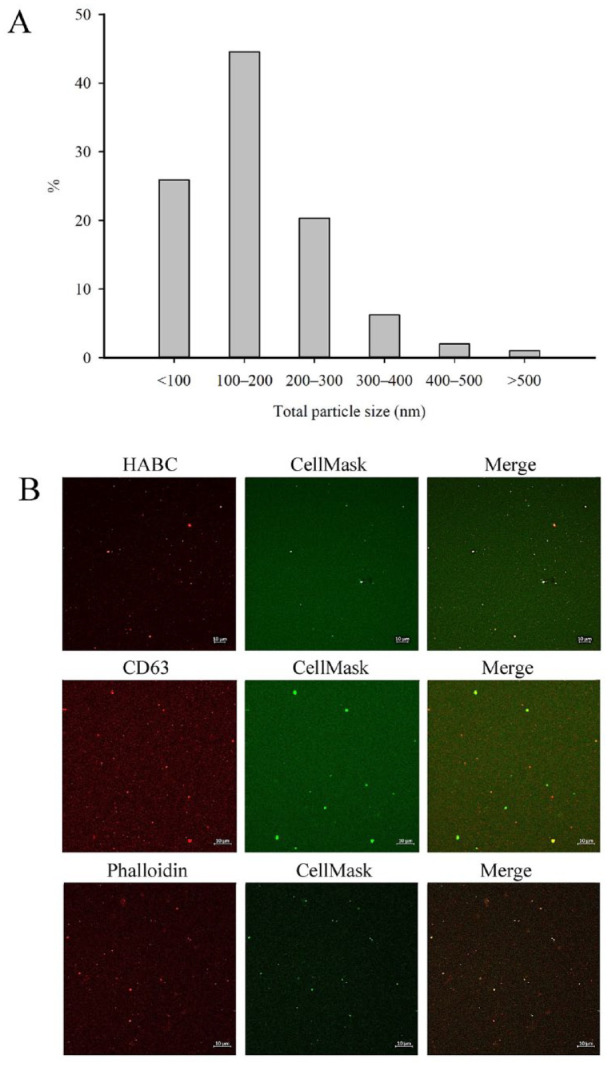
Representative examples of (**A**) size distribution of particles (% of total particle numbers) in human synovial fluid (SF) measured by nanoparticle tracking analysis, and (**B**) large SF extracellular vesicles visualized with the CellMask Deep Red plasma membrane stain (pseudocolored green) and Alexa Fluor-labeled hyaluronic acid binding complex (HABC), tetraspanin CD63, or phalloidin (pseudocolored red) by confocal microscopy. The processing and visualization of the samples were based on previous studies.^4,5^

SF and plasma subsamples were stained with the CellMask Deep Red plasma membrane stain (Life Technologies, Eugene, OR, USA) together with the Alexa Fluor 568-labeled HA binding complex^19^ and placed in eight-well ibidi chambers (ibidi GmbH, Gräfelfing, Germany). The size distribution of SF EVs and the functionality of the stainings were validated for the patient population and are represented in **Figure 1A** and **B**. Confocal laser scanning microscopy (CLSM) was first performed on unprocessed SFs (*n* = 32) and plasma (*n* = 21) followed by centrifugation of SF samples (*n* = 24) at 1000*g* for 10 min at RT to deplete any cell fragments. We utilized the Zeiss Axio Observer inverted microscope equipped with the Zeiss LSM 800 confocal module (Carl Zeiss MicroImaging GmbH, Jena, Germany).^5^ Image acquisition was performed using the ZEN 2.3 blue edition software (Carl Zeiss MicroImaging GmbH). The area and intensity of the stainings, numerical quantification of EVs, HA-particles, and HA–EVs, and the size distribution of EVs and HA-particles were determined with the ImageJ/Fiji *v*1.53 software (NIH, Bethesda, MA, USA) with various open-source plug-ins. Co-localization of EV and HA fluorescences was determined with the ComDet *v*0.4.2 analysis.

### HA Concentrations

The total HA concentration was determined from unprocessed SF (*n* = 60) and plasma samples (*n* = 21) by using a sandwich-type enzyme-linked sorbent assay (ELSA) as previously described.^5^

### Measurements of Physical Function and Pain

Flexion and extension of each knee joint were measured with standard goniometry.^20^ Physical function measurements included a 30-second chair-stand test, a 4 × 10 m fast-paced walk test, and a 12-step stair-climb test recommended by the Osteoarthritis Research Society International.^21^ Subjective and objective intensity of pain was evaluated with visual analog scale (VAS),^22^ pressure pain thresholds (PPTs),^23^ and pain-related questionnaires (painDETECT^24^ and Western Ontario and McMaster Universities Osteoarthritis Index^25^). Psychological well-being was assessed with the Beck depression and Beck anxiety inventories (BAI).^26^ Not all measurements could be made on all participants due to some patients dropping out, and the questionnaires were not obtained from the controls.

### Statistical Analyses

All statistical analyses were performed using the IBM SPSS *v*27 software (IBM, Armonk, NY, USA). The differences were tested with the Kruskal–Wallis one-way analysis of variance (ANOVA) for 3 study groups, or the Mann–Whitney U test for 2 study groups, and the sex ratios within the groups were compared with the Fisher’s exact test. The effects of diagnosis (control, OA, RA), anatomical location (shoulder, knee), and treatment (centrifugation or not) were analyzed with the generalized linear model (GLM). The Spearman’s correlation coefficient (*r_s_*) was calculated to examine the associations between the measured variables and risk factors for OA and RA (age, BMI).^27^ The effects of sex, another risk factor, were tested with the GLM. *P* < 0.05 was considered statistically significant. The univariate ANOVA, adjusted for age and BMI, was used to investigate the associations of the CLSM variables to pain, physical function, and mental health while controlling for these potentially confounding factors. The supervised discriminant analysis (DA) was performed separately on centrifuged and noncentrifuged samples to assess how clearly the SFs differed from one another based on location/diagnosis and which variables were the most important contributors to the models.

## Results

### General Variables

There were no significant differences in sex ratios, but for substudy I, the average age was the highest in the RA group and the average BMI in the OA group, while for substudy II, the OA group had higher average age and BMI compared to the controls (**Table 1**). There were no significant associations between SF HA concentration and age (*r_s_* = −0.131, *p* = 0.353, *n* = 52) or BMI (*r_s_* = −0.032, *p* = 0.822, *n* = 51). In plasma, HA level correlated significantly with age (*r_s_* = 0.654, *p* = 0.001, *n* = 21) but not with BMI (*r_s_* = 0.381, *p* = 0.089, *n* = 21).

### Effects of Diagnosis and Anatomical Location on Unprocessed SFs

**Figure 2.**
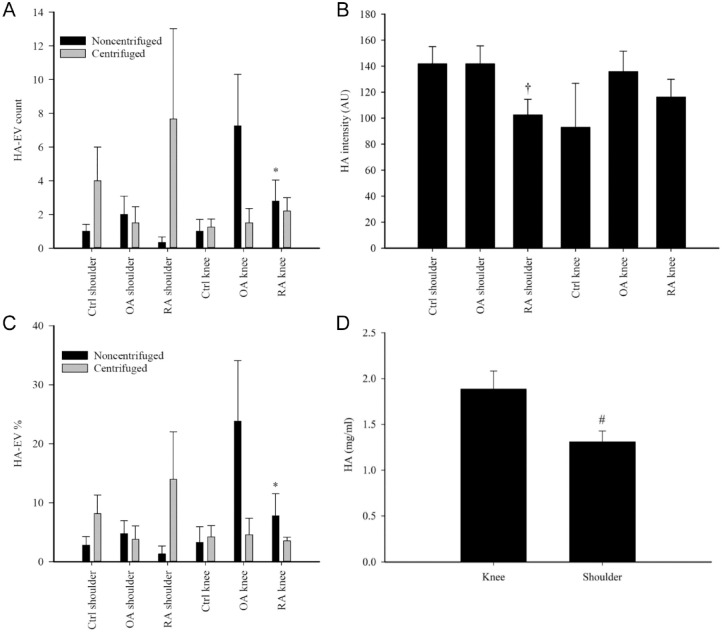
(**A**) Counts and (**C**) percentages of large hyaluronic acid (HA)-containing extracellular vesicles (HA–EVs) according to anatomical location and diagnosis in unprocessed and centrifuged (1000*g* 10 min at RT) synovial fluid (SF) samples, (**B**) intensities of HA fluorescence according to anatomical location and diagnosis in unprocessed SFs, and (**D**) total HA concentrations according to anatomical location in unprocessed SFs (mean + SE). * = statistically significant difference from noncentrifuged OA knees, † = statistically significant difference from Ctrl and OA shoulders (generalized linear model, *p* < 0.05), # = statistically significant difference from knee SFs (Student’s *t*-test, *p* < 0.05), Ctrl = trauma control, OA = osteoarthritis, RA = rheumatoid arthritis, AU = arbitrary unit.

Diagnosis did not affect the SF total HA concentration (GLM, *p* = 0.985, *n* = 12–26/group). In knees, HA–EV count and % of all EVs were lower in RA SF than in OA SF (*p* = 0.023–0.027, *n* = 4–5/group; **Fig. 2A** and **C**). In addition, RA shoulders had a lower intensity of HA fluorescence compared to control and OA shoulders (*p* = 0.029, *n* = 3–4/group; **Fig. 2B**).

**Figure 3.**
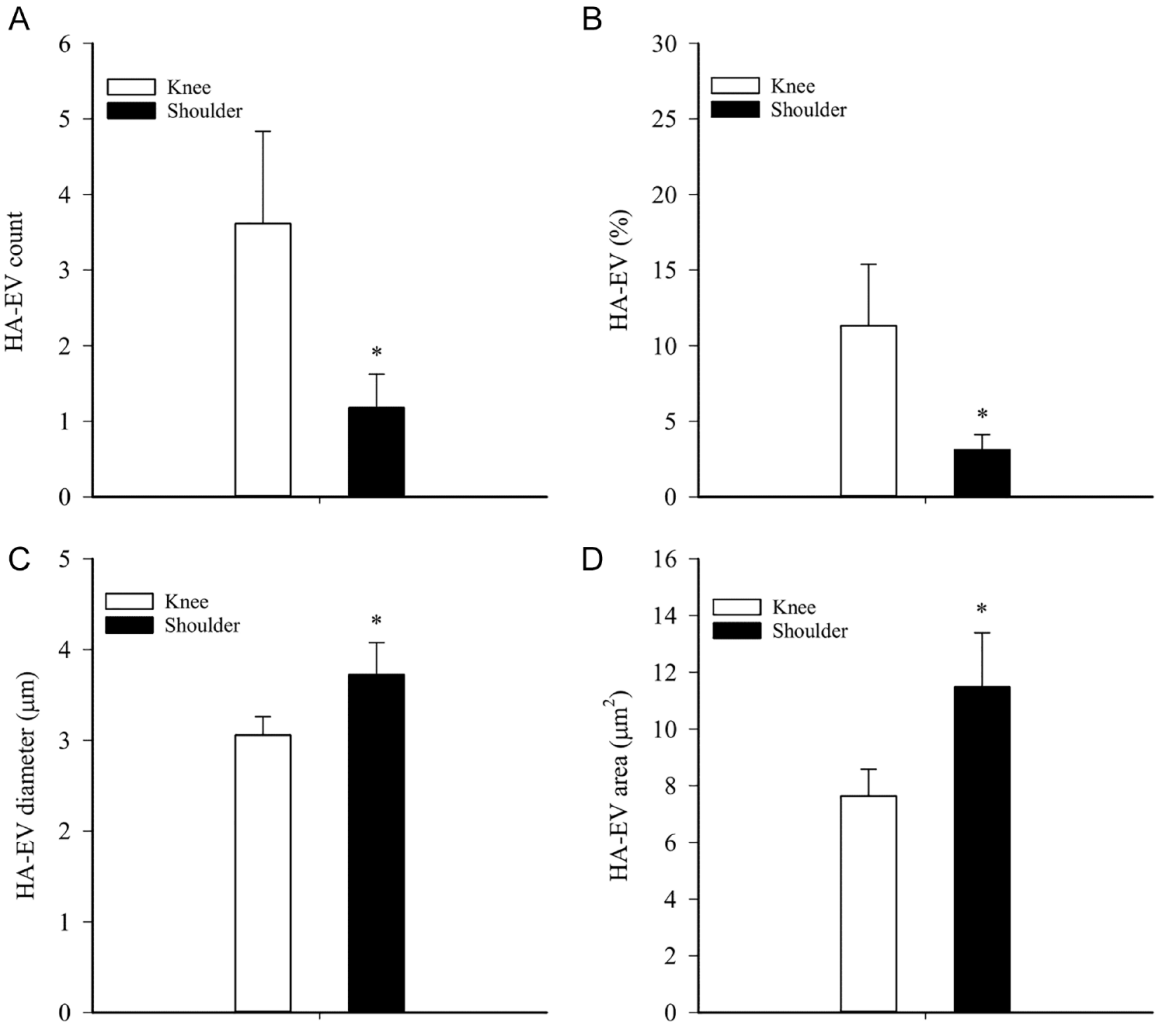
Effects of anatomical location on the characteristics of large hyaluronic acid (HA)-containing extracellular vesicles (HA–EVs) in unprocessed synovial fluids (SFs) of orthopedic patients with traumatized joints, osteoarthritis, and rheumatoid arthritis (mean + SE). (**A**) HA–EV count, (**B**) HA–EV %, (**C**) HA–EV diameter, and **(D**) HA–EV area, * = statistically significant difference from knee SFs (generalized linear model, *p* < 0.05).

There was a trend toward higher total HA concentrations in knee SF compared to shoulder SF (GLM, *p* = 0.080) that was statistically significant in a pair-wise comparison (Student’s *t*-test, *p* = 0.016, *n* = 36 and 16; **Fig. 2D**). HA–EV count and % were lower in shoulder SF (GLM, *p* = 0.013–0.018, *n* = 13 and 11), but their diameter and area were higher (*p* ≤ 0.0005–0.001, *n* = 10 and 7; **Fig. 3**).

**Figure 4.**
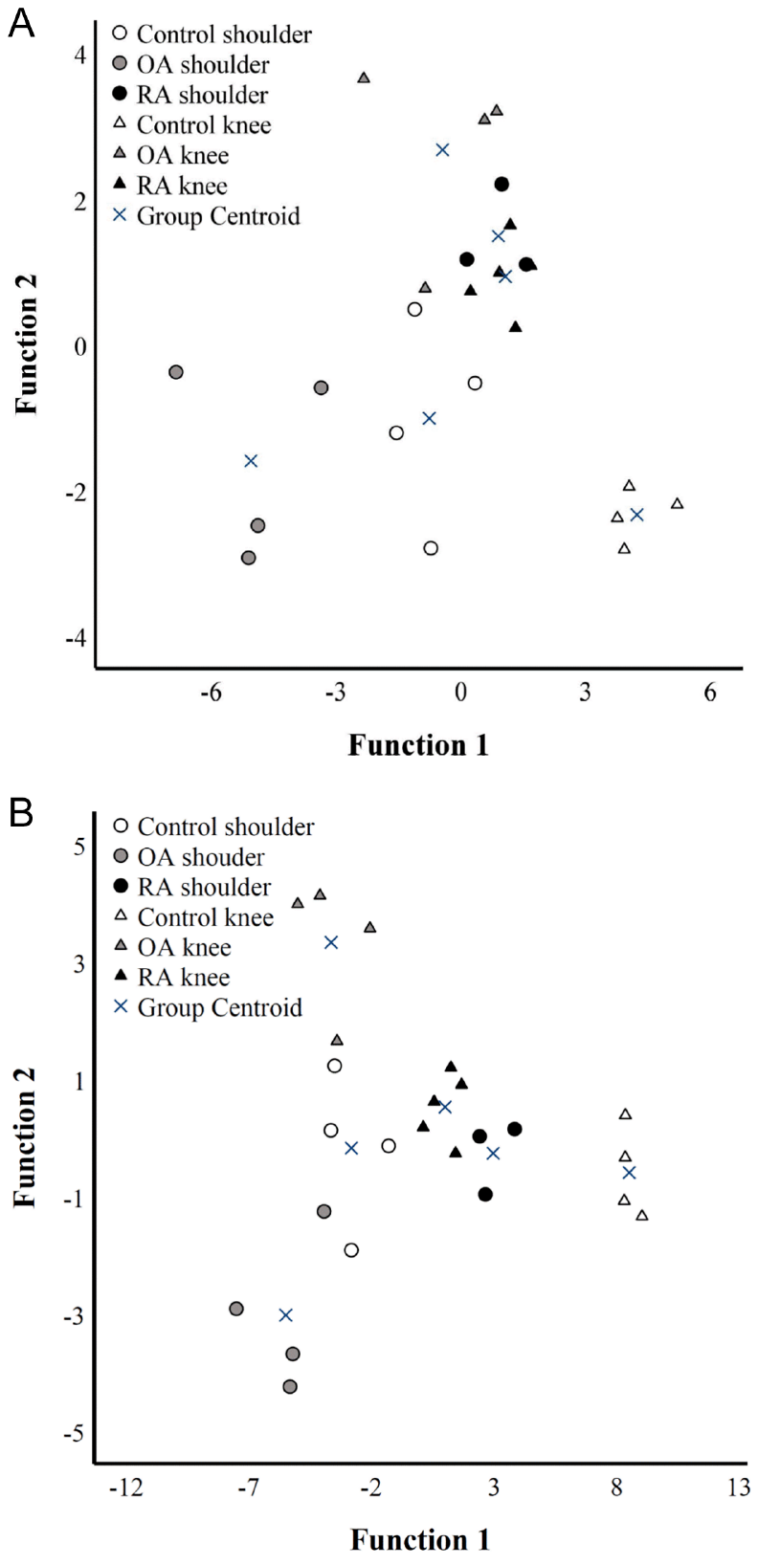
Discriminant analysis of confocal microscopy data from (**A**) unprocessed and (**B**) centrifuged (1000*g* 10 min at RT) shoulder and knee joint synovial fluids. Patient groups included traumatized joints (Control), osteoarthritis (OA), and rheumatoid arthritis (RA). The discriminant function 1 on the x-axis explained 53.2% (**A**) or 77.4% (**B**) of the variance in the dataset and separated especially control knees from OA shoulders. Both analyses classified 95.8% of the samples correctly into their respective location/diagnosis group. White symbols = control, gray symbols = OA, black symbols = RA.

The supervised DA classified 95.8% of the unprocessed samples into their correct study group based on location/diagnosis (**Fig. 4A**). One OA knee was misclassified among RA shoulders. The most important contributors to the model were the intensity of EV fluorescence and the counts of EVs in the size categories of <1 μm and 1–2 μm. The discriminant function 1 (on the x-axis) explained 53.2% of the variance in the dataset and separated especially control knees from OA shoulders. OA knees, RA knees, and RA shoulders were separated from the other location/diagnosis groups by the discriminant function 2 (on the y-axis) explaining 21.9% of the variance.

### Effects of OA on Plasma HA and EVs and Connections to Functionality and Pain

Knee OA was associated with increased plasma HA concentrations compared to controls (control: 62 ± 9, OA: 137 ± 23 ng/ml, Mann–Whitney U test, *p* = 0.011, *n* = 10 and 11). The results of plasma CLSM remained unaffected by diagnosis (*p* > 0.05).

**Figure 5.**
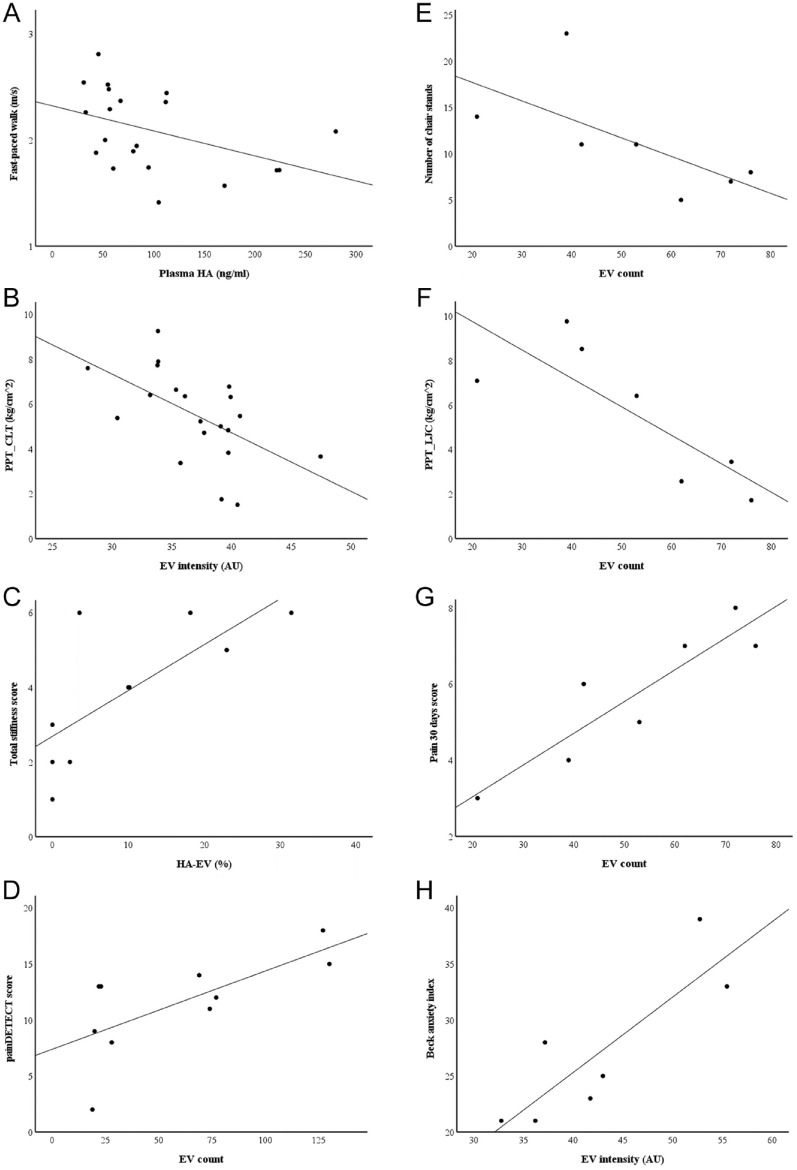
Scatter plots depicting the interrelationships between selected confocal microscopy variables in plasma (**A**)–(**D**) or synovial fluid (**E**)–(**H**) and physiatric measurements. (**A**) plasma hyaluronic acid (HA) and speed of fast-paced walk (*r_s_* = −0.522, *p* = 0.018), (**B**) intensity of extracellular vesicle (EV) fluorescence and pressure pain threshold (PPT) on lateral tibial condyle (CLT) (*r_s_* = −0.565, *p* = 0.009), (**C**) % of HA-containing EVs (HA–EVs) and total joint stiffness (*r_s_* = 0.800, *p* = 0.005), (**D**) EV count and painDETECT score (*r_s_* = 0.681, *p* = 0.030), (**E**) EV count and number of chair-stands (*r_s_* = −0.811, *p* = 0.027), (**F**) EV count and PPT on lateral joint capsule (LJC) (*r_s_* = −0.857, *p* = 0.014), (**G**) EV count and 30d pain score (*r_s_* = 0.901, *p* = 0.006), and (**H**) EV intensity and Beck anxiety inventory (*r_s_* = 0.847, *p* = 0.016), AU = arbitrary unit.

Based on the Spearman’s correlations, plasma HA concentrations showed positive associations with the duration of stair-climb test and current and worst VAS pain, while the associations were negative for the angle of extension, speed of fast-paced walk (**Fig. 5A**), and PPTs on lateral joint capsule. There were also negative associations between the intensity of EV fluorescence and PPTs (lateral joint capsule, medial and lateral tibial condyles; **Fig. 5B**). Stiffness in the morning or total stiffness positively correlated with HA–EV count and % (**Fig. 5C**) and inversely with the diameter and area of these particles. There were positive correlations between EV counts and impairment of total physical function and painDETECT scores (**Fig. 5D**). The areas of HA-particles showed a positive association with PPTs (lateral joint capsule) and a negative correlation with worst pain scores.

Regarding SF, the Spearman’s correlations between EV counts and numbers of chair-stands (**Fig. 5E**) and PPTs (lateral joint capsule, lateral tibial condyle) were negative (**Fig. 5F**). The correlations between EV counts and current pain scores, 30d pain scores (**Fig. 5G**), and impairment of total physical function were positive. The intensity of EV fluorescence positively correlated with BAI (**Fig. 5H**) and inversely with PPTs (*rectus femoris* muscle). The area of EVs inversely correlated with pain scores while standing and with 30d pain scores. HA–EV count showed a positive association with VAS pain, and HA–EV% was inversely linked to night-time pain. Diameter and area of HA–EVs positively correlated with the duration of stair-climb test and negatively with the speed of fast-paced walk. The area of HA-particles positively correlated with PPTs (medial joint capsule, lateral tibial condyle) and negatively with pain scores while standing. In addition, the diameters of EVs and HA-particles had inverse associations to pain scores while standing.

With the univariate ANOVA, we further tested the most interesting of these associations with adjustment for confounding factors, age and BMI. In plasma, we observed that EV counts associated with painDETECT scores (*R*^2^ = 0.640, *F* = 8.951, *p* = 0.024, *n* = 10) and, for SF, EV counts associated with 30d pain scores (*R*^2^ = 0.904, *F* = 17.253, *p* = 0.025, *n* = 7), intensity of EV fluorescence with BAI (*R*^2^ = 0.824, *F* = 11.367, *p* = 0.043, *n* = 7), and area of HA-particles with PPT on lateral tibial condyle (*R*^2^ = 0.870, *F* = 13.173, *p* = 0.036, *n* = 7), independent of age and body adiposity.

### Effects of Preanalytical Processing

**Figure 6.**
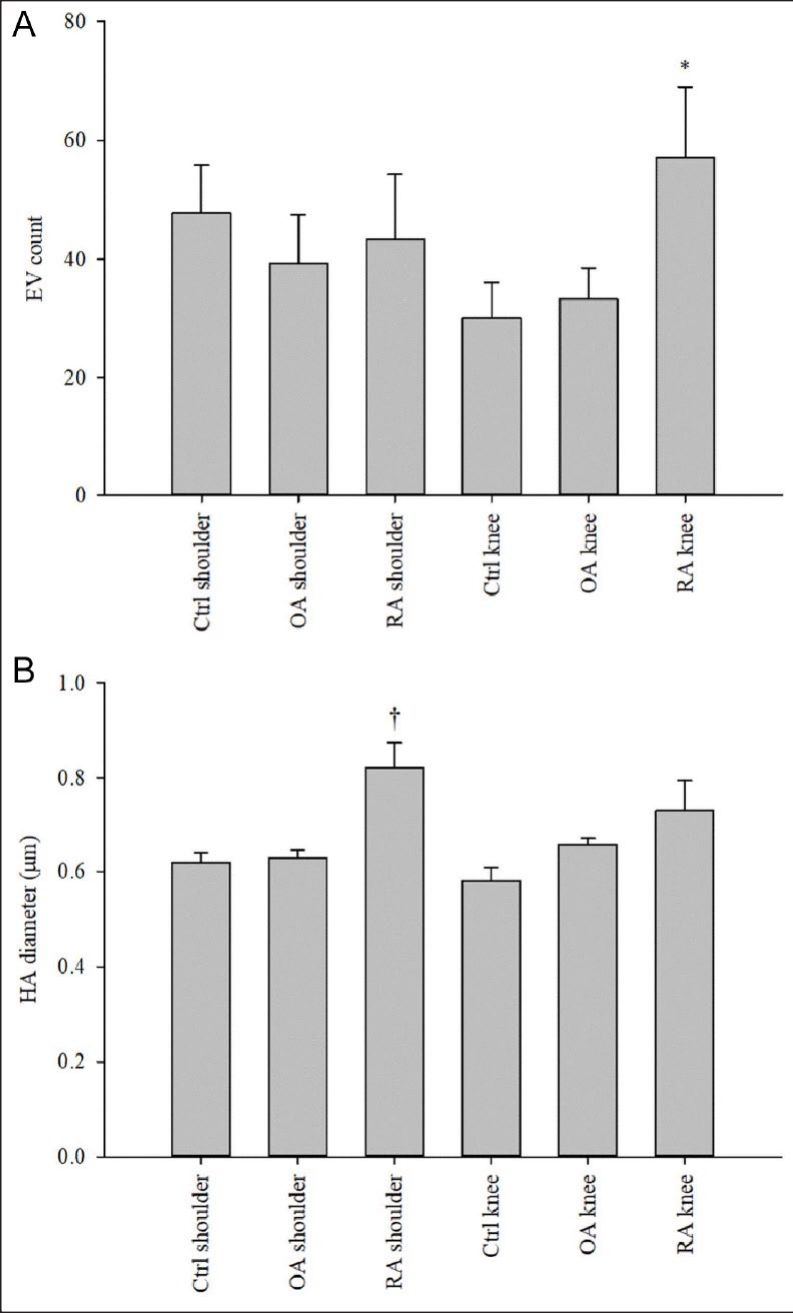
(**A**) Counts of extracellular vesicles (EVs) and (**B**) diameters of hyaluronic acid (HA)-particles according to anatomical location and diagnosis in centrifuged synovial fluids (1000*g* 10 min at RT, mean + SE). * = statistically significant difference from Ctrl and OA knees, † = statistically significant difference from Ctrl and OA shoulders (generalized linear model, *p* < 0.05), Ctrl = trauma control, OA = osteoarthritis, RA = rheumatoid arthritis.

Centrifugation at 1000*g* for 10 min at RT increased the % of EVs with a diameter of 1 to 2 μm compared to the unprocessed samples (GLM, *p* = 0.039, *n* = 24/treatment). It also increased the HA-particle count and intensity of HA fluorescence but decreased the diameter and area of HA-particles (*p* ≤ 0.0005–0.05, *n* = 24/treatment; Suppl. Fig. S1), in agreement with the changes in their absolute and % size distribution (*p* = 0.027–0.048, *n* = 24/treatment; Suppl. Fig. S2). HA–EV count and % were elevated in shoulder SF due to centrifugation (*p* = 0.016–0.023, *n* = 11/treatment). Moreover, EV counts increased after centrifugation in RA SF compared to control and OA SFs but only in knees (*p* = 0.028, *n* = 4–5/group; **Fig. 6A**) and, in shoulders, HA-particle diameters were elevated in RA SF (*p* < 0.0005, *n* = 3–4/group; **Fig. 6B**).

The supervised DA of the centrifuged SFs classified 95.8% of the samples into correct location/diagnosis (**Fig. 4B**). One control shoulder was misclassified among RA knees. The most important contributors to the model included the counts of EVs <1 μm and 4 to 5 μm of diameter and total EV count. Function 1 explaining 77.4% of the variance in the dataset separated especially control knees from OA shoulders. Function 2 discriminated especially OA knees from OA shoulders and explained 12.3% of the variance.

## Discussion

This study focused on the examination of HA-particles in their natural environments and on the effects of preanalytical processing on SF HA characteristics. Regarding SF and plasma EVs, we concentrated on the larger membrane-bound particles that are often discarded by isolation methods, which can hinder the investigation of their biological functions. The main findings were as follows: (1) OA and RA SFs can be distinguished from trauma joints based on the distinctive profiles of their HA-particles and large EVs, (2) there are differences in the SF HA and EV characteristics between shoulder and knee joints, (3) EV counts in plasma and SF associate with specific pain and functional parameters, and (4) low-speed centrifugation causes significant alterations in the features of HA-particles and EVs, thus, complicating their examination in the original state.

EVs in human SF are heterogenous in size, and there are at least two major subpopulations with diameters of <300 nm and >700 nm.^4,5,7^ The numbers of smaller EVs (exosomes and microvesicles) can be analyzed with, for example, nanoparticle tracking analysis, while CLSM is a useful method to characterize larger EVs that are often ignored in research settings due to isolation protocols designed for smaller EVs. Thus, the knowledge on the properties and function of large EVs in biofluids is mostly lacking. As examples of larger EVs having potential medical applications in disease monitoring and unraveling pathogenic processes, it is known that platelet-poor plasma of patients with systemic lupus erythematosus contains large EVs (700–3000 nm) with bound IgG and mitochondrial proteins, probably resulting from apoptosis,^28^ while RA SF contains large pro-inflammatory macromolecular structures (700–3000 nm) with both immune complexes and EVs.^7^ This suggests that large EVs are not solely apoptotic bodies but autoantigen-expressing elements capable of perpetuating the formation of inflammatory immune complexes. The presence of large EVs is not limited to autoimmune diseases, but they have been documented in OA SF,^4,5^ and they also include cancer cell-derived EVs.^6^ To accommodate the MISEV guidelines for studies on EVs,^29^ these structures were shown to have intact lipid membranes and to stain with membrane tetraspanin CD63 and phalloidin that binds cytosolic actin^5^ (**Fig. 1B**), and they lacked staining of nuclei by NucBlue. Thus, we are fairly confident that the population of large EVs detected in trauma/arthritic SF and in control/OA plasma did not mainly consist of large lipid particles, apoptotic bodies, or cell fragments.

Based on the DA of noncentrifuged SFs, shoulder and knee samples were clearly separated for both control and OA. In contrast, shoulder and knee RA SFs aligned together. The intensity of EV fluorescence and the counts of EVs <2 μm in diameter were the most important contributors to the model. HA–EVs were generally more abundant in knee SFs compared to shoulder SFs, and there was also a tendency toward higher total HA levels in knees. These site-specific differences may be associated with the very dissimilar weight-bearing properties, as the knee joint is under considerable weight stress, and even more so in the obese OA population (BMI 34.9 kg/m^2^). Different loading properties between the joints as a plausible reason for the higher HA concentration in the knee are supported by the fact that HA secretion is stimulated by cyclic compressive loading and joint movement.^30,31^ Proteoglycan synthesis did not previously differ between humeral head, glenoid, and femoral cartilages,^32^ while the proportions of polyunsaturated fatty acids (PUFAs), that are constituents of surface-active phospholipids and function in boundary lubrication, were higher in arthritic shoulder than knee SF.^16^ The shoulder as a ball-and-socket joint has much more extreme positional mobility than the biomechanically complex hinge-like joint of the knee.^33,34^ While the shoulder joint can also experience relatively high loads due to its small surface area, especially when lifting and supporting weight, the knee joint is under much more weight-bearing on a more constant basis. Thus, it is reasonable that the shoulder joint would need especially lubrication and the knee joint would have, in addition to lubrication, high demands for load-bearing and shock absorption. Consequently, it can be speculated that the higher HA concentrations in the knee joint could provide better shock absorption,^35^ while the elevated PUFA proportions in the shoulder joint may be associated with appropriate lubrication properties.^36^ Obviously, neither HA nor PUFAs are alone responsible for the friction-inhibiting properties of SF, and several models have been introduced on the tight interactions between HA, surface-active phospholipids, and other components of SF in the promotion of boundary lubrication for articular cartilage.^9^

In the univariate GLM, HA–EV count and % stood out as variables that differentiated knee SFs between OA and RA. The same variables were recently suggested to have potential as SF biomarkers in equine OA.^18^ In the CLSM of unprocessed samples, RA shoulders had lower intensities of HA fluorescence compared to control and OA shoulders, similar to earlier results on knee SFs.^5^ The total SF HA concentrations measured by ELSA did not differ between the diagnoses, even though inflammation has previously increased HA production by synoviocytes,^37^ and OA and RA SFs have displayed lower levels of high-molecular-weight HA.^9^ Obviously, RA patients were undergoing immunosuppressive medication that could not be discontinued for the study due to ethical reasons, and it is possible that the dampened inflammatory status could have affected their HA response compared to the observed increase in the plasma HA of OA patients, agreeing with previous literature.^38^ Neither was the SF HA level associated with age in the present experiment. This is different from a previous study by Temple-Wong *et al.*,^39^ who found an age-associated change in HA concentrations that was −10.5% per decade. HA concentration and quality were more strongly associated with age than with the grade of joint degradation. With the wide age distribution of the subjects (19–85 years), we could have expected significant correlations in the HA-related variables, but as the younger patients were arthritis-free in contrast to the older individuals, it is possible that the changes caused by OA or RA masked the age-related association compared to a hypothetical situation of all subjects having non-arthritic joints.

Particular confocal microscopy parameters in plasma and SF correlated with different pain categories, joint stiffness, functional limitations, and mental health. We further tested the most interesting of these associations with univariate ANOVAs adjusted to the potentially confounding factors, age and BMI, to unravel their possible contribution to statistically significant findings. Plasma EV counts were documented to associate with painDETECT scores, and SF EV counts with 30d pain scores. Moreover, the intensity of SF EV fluorescence associated with BAI, and the area of HA-particles with PPT on lateral tibial condyle. It was previously documented that serum EV subpopulations with specific tetraspanins and platelet markers can be significant predictors of OA pain,^40^ while in rodent models, mesenchymal stem cell-derived EVs may reduce OA-related pain behavior.^41,42^ The present results indicate that plasma and SF EVs can be significant predictors of OA symptoms, independent of age and adiposity, and suggest that their implications in OA pathophysiology warrant further study.

Low-speed centrifugation at 1000*g* resulted in alterations in the characteristics of HA-particles and EVs in human SF, like previously documented for equine SF.^18^ This study demonstrated increases in HA-particle counts and fluorescence with simultaneous reductions in their average diameter and area, and the size distribution of HA also changed toward smaller particles. This suggests centrifugation-induced fragmentation of larger HA-complexes into smaller molecules. Moreover, RA knee SF showed elevated EV counts in centrifuged samples compared to control and OA knees. Increased EV numbers are a common finding in RA SF,^2^ but it is plausible that the elevated counts of large EVs in this study were a result of sample processing. It is known from previous experiments that centrifugation at 18,890*g* can lead to aggregation of platelet-derived EVs^43^ and that the centrifugation speed of 1500*g* can yield 10- to 15-fold higher counts of platelet-derived microparticles compared to 5000*g*, presumably due to higher levels of cell debris, precipitates, and irrelevant cellular elements.^44^ To the best of our knowledge, previous data on the effects of centrifugation on HA characteristics are limited. If the goal was to examine the features of SF as authentically as possible, even low-speed centrifugation should be avoided. This would also enable the investigation of larger EVs that are usually removed by ultracentrifugation protocols.^6^ Obviously, this can be supplemented by subsamples with various centrifugation regimes, which would necessitate adequate sample volumes. Still, it seems clear that data from centrifuged and noncentrifuged SFs reflect different issues within the same samples, and assessing both types would add to our knowledge on membrane-bound particles in the pathogeneses of joint diseases.

Owing to the small sample size in this study, the patient groups could not be matched for age or BMI, while the sex ratio did not differ between diagnoses. Thus, the age- and adiposity-related differences between the groups limit the obtained data, and the results are of preliminary nature, although the univariate ANOVA adjusted for the confounding factors was able to overcome some of these issues. Research on the significance of EVs in joint diseases is still in its early stages. At present, it is important to accurately describe the EV populations that are secreted by arthritic tissues and select those variables that are the most promising for future studies that will concentrate on the clinical significance and potential therapeutic applications. As OA and RA diagnoses routinely rest on a solid base of radiology and immunochemistry, it is obvious that in clear-cut cases, EV or HA analyses would not be directly applicable to diagnostics. However, CLSM could yield additional clinically applicable data to assess, which cases would have the most potential of developing debilitating symptoms and be selected for early-stage therapies and rehabilitation. Methodologically, CLSM is useful in characterizing larger EVs bearing in mind that a significant number of small exosomes remains undetected. While it can also give an estimate of the size of HA-particles, it is obviously not equivalent to the measurements of high- and low-molecular-weight HA. However, the strength of CLSM is in its ability to visualize the hitherto mostly neglected population of large EVs in their undisturbed state.

The biological functions of large EVs still mostly remain uninvestigated, as they are routinely discarded by the often-used isolation protocols.^6^ This seems to be an oversight, as in autoimmune diseases, large EVs form immune complexes, act as sites of autoantigen expression, and can associate with plasma pro-inflammatory cytokines.^7,28^ They have been suggested to promote immune cell activation and inflammation, for instance, the production of leukotrienes by neutrophils, and the results of this study propose an association with joint pain. Future research is required to verify the physiological roles of large EVs in autoimmune and other joint diseases and to standardize their isolation and quantification protocols in different biofluids. These structures, such as HA–EVs, have great potential as disease biomarkers, therapeutic targets, and vehicles for drug delivery, and the combination of EVs with high-molecular-weight HA is considered a promising application for cartilage repair and regeneration.^13^ In addition to introducing extrinsic HA or HA–EV combinations into the joint space, understanding the production, function, and clearance of large EVs would be the first step toward designing interventions that could promote or attenuate the intrinsic HA–EV production. Regarding disease pathogenesis, large EVs could represent an additional mechanism of intra-articular cartilage–synovium communication, and provide a tool to understand the pathogenesis of OA/RA symptoms and to design new prognostic and therapeutic options.

## Supplemental Material

sj-docx-1-car-10.1177_19476035241247659 – Supplemental material for Hyaluronic Acid and Large Extracellular Vesicles (EVs) in Synovial Fluid and Plasma of Patients With End-Stage Arthritis: Positive Association of EVs to Joint Pain Supplemental material, sj-docx-1-car-10.1177_19476035241247659 for Hyaluronic Acid and Large Extracellular Vesicles (EVs) in Synovial Fluid and Plasma of Patients With End-Stage Arthritis: Positive Association of EVs to Joint Pain by Anne-Mari Mustonen, Janne Capra, Sanna Oikari, Laura Säisänen, Lauri Karttunen, Petro Julkunen, Petri Lehenkari, Antti Joukainen, Antti Jaroma, Tommi Paakkonen, Tommi Kääriäinen, Heikki Kröger and Petteri Nieminen in CARTILAGE

sj-docx-2-car-10.1177_19476035241247659 – Supplemental material for Hyaluronic Acid and Large Extracellular Vesicles (EVs) in Synovial Fluid and Plasma of Patients With End-Stage Arthritis: Positive Association of EVs to Joint Pain Supplemental material, sj-docx-2-car-10.1177_19476035241247659 for Hyaluronic Acid and Large Extracellular Vesicles (EVs) in Synovial Fluid and Plasma of Patients With End-Stage Arthritis: Positive Association of EVs to Joint Pain by Anne-Mari Mustonen, Janne Capra, Sanna Oikari, Laura Säisänen, Lauri Karttunen, Petro Julkunen, Petri Lehenkari, Antti Joukainen, Antti Jaroma, Tommi Paakkonen, Tommi Kääriäinen, Heikki Kröger and Petteri Nieminen in CARTILAGE
