# Supplementary figures and images for: Hyaluronic Acid and Large Extracellular Vesicles (EVs) in Synovial Fluid and Plasma of Patients With End-Stage Arthritis: Positive Association of EVs to Joint Pain
Source: Cartilage. 2024 May 10;16(2):169–80. doi: 10.1177/19476035241247659 (PMC11569565; doi:10.1177/19476035241247659)

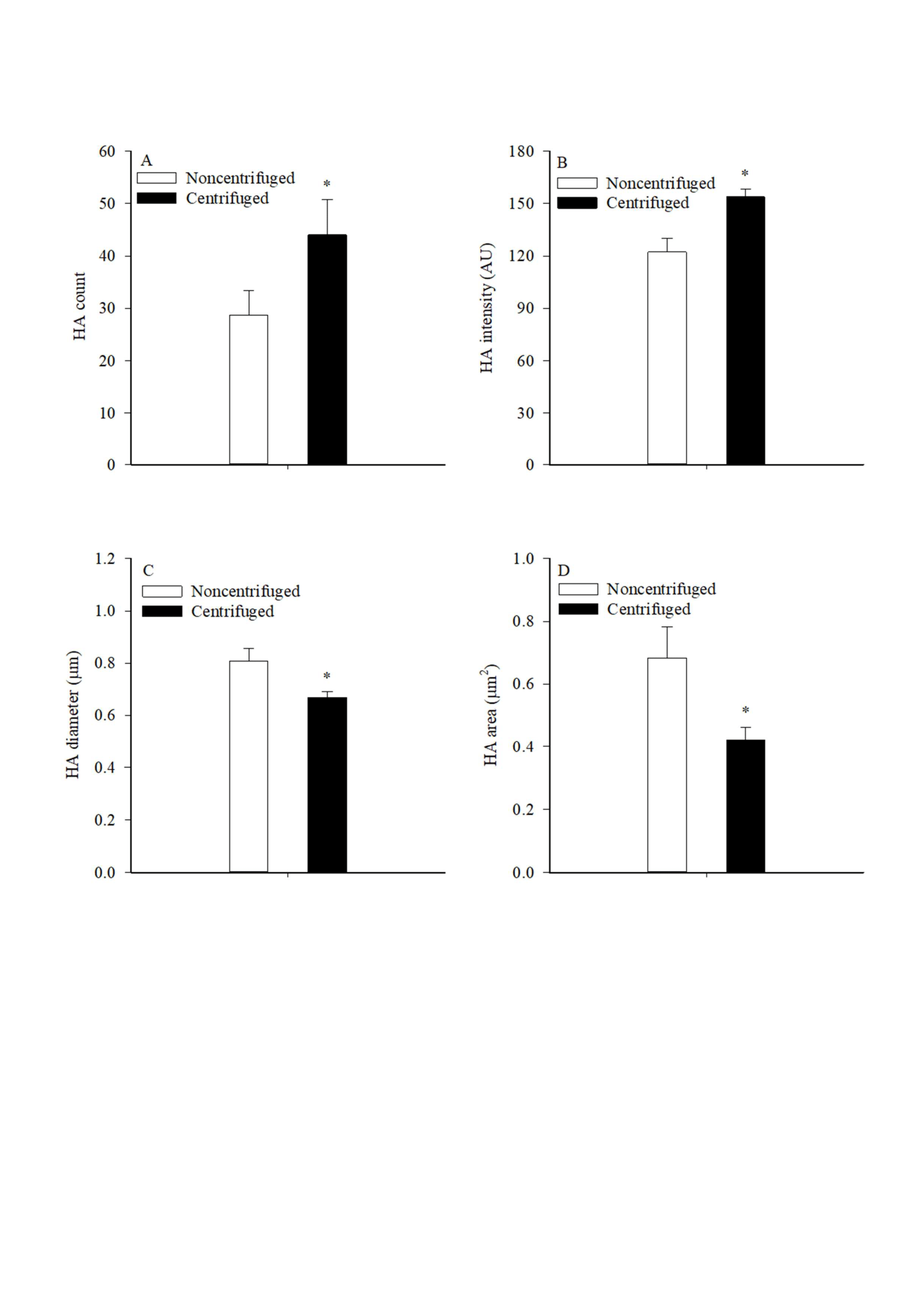

Supplement: sj-docx-1-car-10.1177_19476035241247659 – Supplemental material for Hyaluronic Acid and Large Extracellular Vesicles (EVs) in Synovial Fluid and Plasma of Patients With End-Stage Arthritis: Positive Association of EVs to Joint Pain [file sj-docx-1-car-10.1177_19476035241247659.docx]

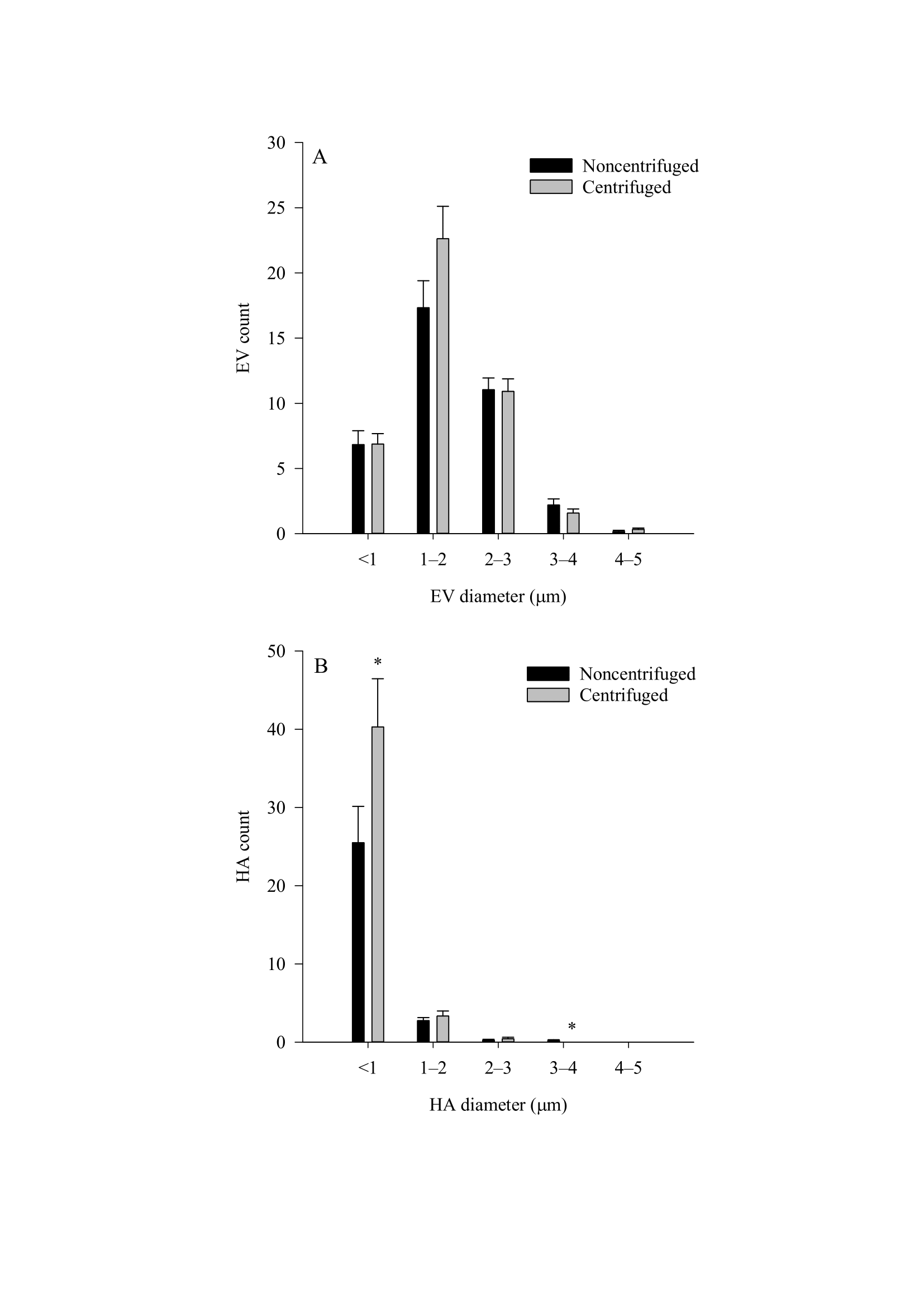

Supplement: sj-docx-2-car-10.1177_19476035241247659 – Supplemental material for Hyaluronic Acid and Large Extracellular Vesicles (EVs) in Synovial Fluid and Plasma of Patients With End-Stage Arthritis: Positive Association of EVs to Joint Pain [file sj-docx-2-car-10.1177_19476035241247659.docx]
